# Hydration Mechanisms of Gelled Paste Backfills for Potash Mines Using Lime as a Gel Material

**DOI:** 10.3390/gels10120832

**Published:** 2024-12-18

**Authors:** Rongzhen Jin, Xue Wang, Xuming Ma, Huimin Huo, Siqi Zhang, Jiajie Li, Wen Ni

**Affiliations:** 1 Key Laboratory of Resource-Oriented Treatment of Industrial Pollutants, School of Civil and Resource Engineering, University of Science and Technology Beijing, Beijing 100083, China; kimyeongjin@icloud.com (R.J.); wangxue@ustb.edu.cn (X.W.); niwen@ces.ustb.edu.cn (W.N.); 2 Solid Waste and Chemicals Management Center of the Ministry of Ecology and Environment of China, No.1 Yuhui South Road, Chaoyang District, Beijing 100029, China

**Keywords:** potash mine, gelled filling, lime, microanalysis, brine water

## Abstract

This paper investigates the flow performance and mechanical properties of underground gelled filling materials made from potash mine tailings, using lime as a gel. It demonstrates the feasibility of using lime as a gel, potash mine tailings as aggregate, and replacing water with potash mine tailings to create filling materials that meet design requirements for flow and compressive strength. The role of lime in the hardening process is explored through X-ray diffraction, scanning electron microscopy with energy-dispersive X-ray spectroscopy, thermogravimetric analysis, and infrared analysis. Results show that hydration products vary with lime dosage. With 9% lime (L9), the products are primarily ghiaraite (CaCl_2_·4H_2_O) and carnallite (KMgCl_3_·6H_2_O); with 5% lime (L5), tachyhydrite (CaMg_2_Cl_6_·12H_2_O) predominates, along with minor amounts of antarcticite (CaCl_2_·6H_2_O) and korshunovskite (Mg_2_Cl(OH)_3_·4H_2_O); and with 2.6% lime (L2.6), the products include tachyhydrite, ghiaraite, bischofite (MgCl_2_·6H_2_O), and korshunovskite. These hydration products form a dense, interwoven structure, enhancing the strength of the filling material. This study offers a theoretical foundation for using lime gel as a filling material in potash mining, with significant implications for sustainable mining practices.

## 1. Introduction

China, the world’s largest consumer of potash fertilizer, has long faced a chronic shortage of potash resources, relying heavily on imports to meet its agricultural needs. Due to the uneven distribution of global potash reserves and the concentration of production in just a few countries, major potash-consuming nations like China are highly dependent on imports [1]. In 2017, China’s potash fertilizer imports (in terms of KCl) reached 7.61 million tons, marking an 11.6% increase from 2016 [2]. Projections for 2018 indicated an even higher import volume of 8–9 million tons, demonstrating the country’s substantial and growing demand for potash. To address this issue, the “13th Five-Year Plan” for China’s potash industry development encourages enterprises to expand overseas operations under the “Belt and Road” initiative, focusing on the exploration and utilization of global potash resources [3]. In line with this strategy, the National Development and Reform Commission (NDRC) proposed the “three 1s” development model: one-third of potash supply from domestic production, one-third from imports, and one-third from overseas production bases, aiming to ensure China’s food security [4].

Since the early 2000s, China has been investing in overseas potash resources, with more than 30 projects across countries like Laos, Canada, the Republic of the Congo, and Kazakhstan, amounting to over CNY 10 billion in total investment [5,6]. Despite these efforts, the potash mining industry faces significant challenges in waste management. The complex mining process generates large quantities of potash tailings and waste liquids, which, if improperly handled, pose safety risks, incur high storage costs, and can severely damage both the ecological and social environments [7,8]. The disposal of these by-products has become a critical issue for the sustainable development of potash mining, with companies urgently seeking effective solutions for managing tailings and waste liquids. Addressing this problem is key to ensuring the long-term viability of China’s potash industry. Gelled paste backfill is an appliable way to solve the problem and has been used in many mines [9,10,11].

MgO and oxy-chloride magnesium cement have been used as a binder in potash mine backfill material, but its high reaction rate causes poor flowability and is unsuitable for long-distance transportation [12,13,14]. Previously, steel slag has been used as a gel for potash mine backfill in our group and has proved to perform well in this area [15,16]. However, in Laos, the steel industry is very small, which makes it hard to obtain the steel slag raw material. Lime, an air-hardening inorganic gel with CaO as its main component, is one of the earliest gels used by humans and can be widely found. Specifically, it can be easily found around the potash mine we studied. It has been widely employed in underground cementing and filling operations in both ferrous and nonferrous metal mines. For instance, Zheng et al. [17] used lime as a gel, combined with coarse and fine gold mine tailings as aggregates, to prepare a filling material that achieved a maximum strength of 2.03 MPa after 28 days with a lime dosage of 5%. Similarly, Li et al. [18] utilized lime as an activator, adding water-quenching slag, cement clinker, and gypsum to produce a composite cement. When mixed with tailings, this gelled filling material reached a compressive strength of up to 2.03 MPa after 28 days of curing. When mixed with tail sand and water, the compressive strength of the resulting material exceeded 3 MPa after 28 days of curing.

However, most current studies have not explored the use of lime as a gel for underground gelled filling in potash mines. In this study, it was found that potash mine gelled fills prepared with lime as a gel not only met the strength requirements after 28 days but also exhibited excellent flow properties within a certain timeframe. Additionally, the hardening mechanism of the lime-based gel in the gelled fill was investigated using microanalytical techniques, including X-ray diffraction (XRD), scanning electron microscopy with energy-dispersive X-ray spectroscopy (SEM-EDS), thermogravimetric analysis (TG-DTA), and Fourier-transform infrared spectroscopy (FTIR).

## 2. Results and Discussion

### 2.1. Results of CPBs with Different Lime Dosages and Solid Concentrations

#### 2.1.1. Fluidity

**Figure 1:**
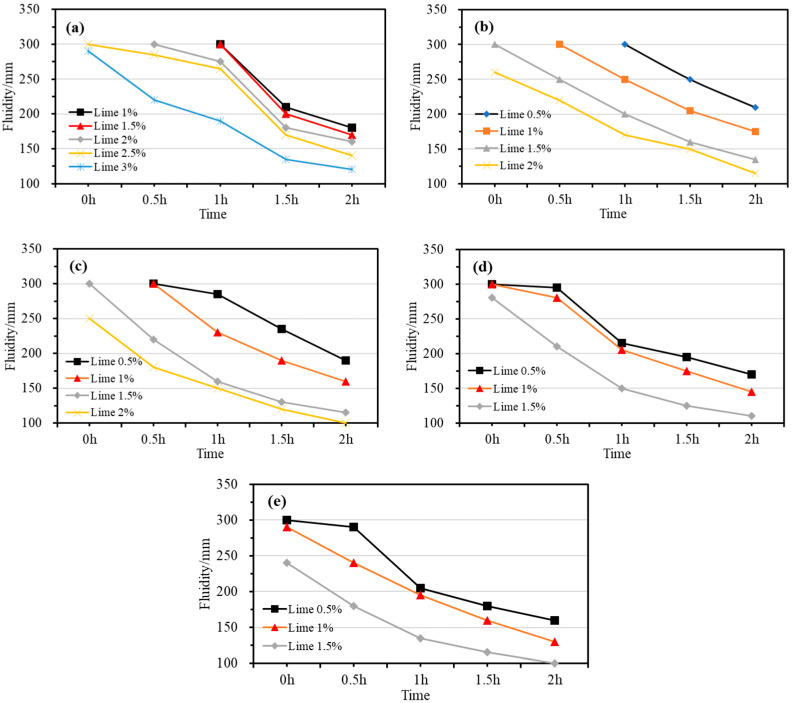
Fluidity results of CPBs with different lime dosages at solid concentrations of (**a**) 55%; (**b**) 60%; (**c**) 65%; (**d**) 70%; (**e**) 75%.

In mining engineering, early fluidity is a crucial indicator of the workability and transport performance of filling materials. Typically, a fluidity of over 220 mm allows for gravity transportation through pipelines, while a fluidity of over 180 mm enables pumping. The fluidity of CPBs was tested every 0.5 h, as shown in Figure 1, which illustrates the change in flowability over time for slurry prepared under different proportioning conditions in the lime dosing test. The results indicate that when lime is used as a gel for gelled filling materials in potash mines, the fluidity decreases over time. This suggests that lime reacts with the brine water, causing the slurry to gradually thicken as it hardens into a filler, leading to increased strength. Compared to steel slag, lime causes a more significant reduction in flowability [15,16], even though its dosage is lower, highlighting its higher reactivity with the brine water.

Figure 1a illustrates the flowability trend of the prepared filling slurry over time, with a fixed solid concentration of 55% and varying lime dosages (1–3%). As the lime dosage increases, the flowability decreases over time. For the test groups with lime dosages of 2.5% and 3%, the initial fluidity values are not shown in Figure 1a due to segregation in the slurry, which prevents accurate measurement of the initial fluidity. This issue also affects Figure 1b,c. When the lime dosage is 1%, the initial flow is 300 mm, but it drops to 170 mm after 2 h. With 3% lime, the initial flow is 290 mm and decreases to 120 mm after 2 h. At 2% lime, the flow initially is 260 mm but starts to decrease after 45 min, reaching 115 mm after 2 h. For slurries with a solid concentration of 65% and lime dosages ranging from 0.5% to 2%, Figure 1c shows that the initial flow is 300 mm at 0.5% lime, which decreases to 190 mm after 2 h. At 2% lime, the initial flow is 250 mm, decreasing to 180 mm after 30 min and 100 mm after 2 h. These results indicate that with 65% solid concentration, the lime dosage should not exceed 2% to maintain acceptable flowability. Figure 1d shows the flow trend for slurries with 70% solid concentration and lime dosages between 0.5% and 1.5%. At 0.5% lime, the initial flow is 300 mm, dropping to 170 mm after 2 h. At 1.5% lime, the initial flow is 280 mm, decreasing to 110 mm after 2 h. Lastly, Figure 1e demonstrates the flow trend for slurries with 75% solid concentration and lime dosages between 0.5% and 1.5%. At 0.5% lime, the initial flow is 300 mm, decreasing to 160 mm after 2 h. At 1.5% lime, the initial flow is 240 mm, dropping to 100 mm after 2 h.

The test results show that the flowability of the filling slurry gradually decreases over time. Additionally, for a constant solid concentration, a higher gel dosage leads to a greater loss of flowability. This is because an increased lime dosage raises the likelihood of free water in the waste liquid reacting with the gel, speeding up the reaction. As a result, the free water content in the slurry decreases, causing the slurry to thicken and its flowability to reduce.

The fluidity results indicate that using lime as a gel in underground filling materials for potash mines requires adjusting the solid concentration based on the lime dosage. To ensure the slurry remains transportable for 1 h, the fluidity must exceed 180 mm within that time. For lime content of 0.5–1%, the solid concentration should be 55–75%; for 1–2%, it should be 55–65%; and for 2–3%, it should be 55% or lower.

#### 2.1.2. Compressive Strength

**Figure 2:**
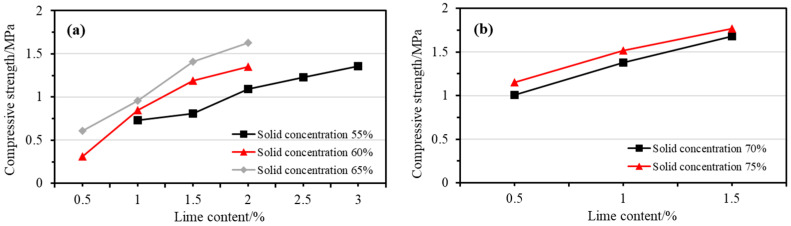
The 28-day compressive strength results of CPBs with different lime dosages at solid concentrations of (**a**) 55%, 60%, and 65%; (**b**) 70% and 75%.

Figure 2 presents the compressive strength test results for the filling slurry prepared under various proportioning conditions in the lime gel dosing test, molded and cured for 28 days. The results show that the compressive strength of the filling block mirrors that of the lime gel dosing test. As seen in the Figure 2, when lime is used as a gel for underground gelled fillings in potash mines, compressive strength increases with higher lime dosages, given the same solid concentration. Additionally, for a fixed lime dosage, increasing the solid concentration also enhances compressive strength.

As shown in Figure 2a, the 28-day compressive strength of the filled blocks increases with higher gel dosages at solid concentrations of 55%, 60%, and 65%. At 55% solid concentration, with 1% lime addition, the compressive strength is 0.73 MPa, below the target of 1 MPa. However, increasing the lime content to 2% raises the strength to 1.09 MPa, and at 3%, it reaches 1.36 MPa, meeting the strength requirement. At 60% solid concentration, 0.5% lime doping results in a compressive strength of 0.31 MPa, which is insufficient. Increasing the lime content to 1.5% raises the strength to 1.19 MPa, and at 2%, it reaches 1.35 MPa, satisfying the strength requirement. At 65% solid concentration, 0.5% lime doping yields 0.61 MPa, still below target, but increasing lime to 1.5% boosts the strength to 1.41 MPa, and at 2%, it reaches 1.63 MPa, meeting the strength requirement.

Figure 2b shows that at 70% and 75% solid concentrations, the compressive strength also increases with lime doping, following a similar trend. At 70% solid concentration, 0.5% lime results in 1.01 MPa, meeting the strength requirement, and 1.5% lime raises it to 1.68 MPa. At 75% solid concentration, 0.5% lime yields 1.15 MPa, while 1.5% increases it to 1.77 MPa. Thus, at both concentrations, lime doping above 0.5% ensures the filling material meets the strength requirements.

The test results show that when lime is used as a gel for underground filling material in potash mines, the required solid concentration depends on the lime dosage. For lime content of 0.5–1%, the solid concentration should be at least 70%; for 1–2%, it should be 60–70%; and for 2–3%, it can be below 60%. These ranges ensure the 28-day compressive strength exceeds 1 MPa. While increasing lime dosage can lower the solid concentration, further testing is needed to determine the exact solid concentration achievable with higher concrete dosages.

In summary, to satisfy both the flow and strength requirements of the filling material—specifically, a flow of 180 mm or more within 1 h and a compressive strength of at least 1 MPa after 28 days—the findings on solids concentration at varying lime dosages are as follows: for lime content of 0.5–1%, the solid concentration should be 70–75%; for 1–2%, it should be 60–65%; and for 2–3%, it can be 55% or below.

Using lime as a gelling material offers several advantages over steel slag and other alternatives. Lime achieves high strength with minimal dosage (only 0.5% to 2%), leading to substantial resource savings. Besides, derived from limestone, a widely available and commonly used industrial raw material, lime is easily accessible. The transportation distance between gel material sources and mines significantly impacts transportation costs. Using lime as a gel enhances resource availability, and lowers environmental carbon emissions, making it a cost-effective and eco-friendly choice.

### 2.2. Results and Analysis on Pastes

#### 2.2.1. Compressive Strength Results

**Figure 3:**
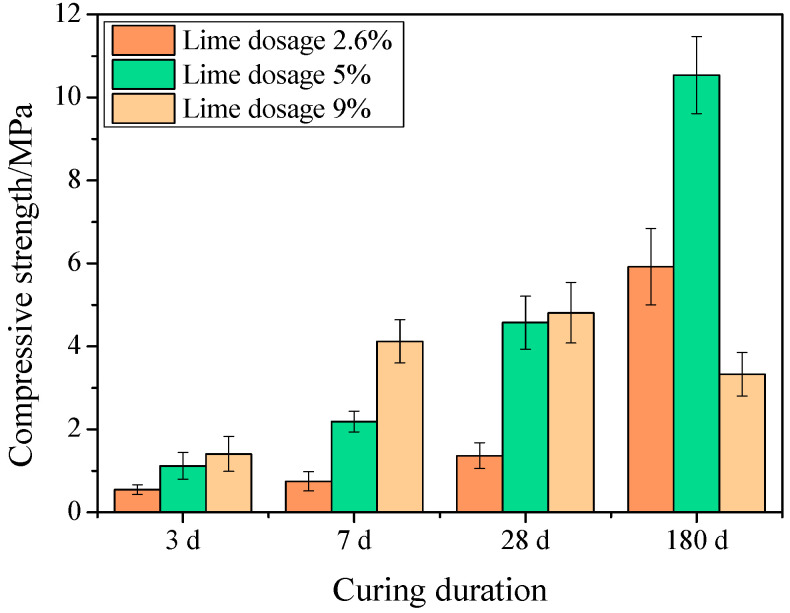
Strength at various ages of specimens with different lime dosages.

Figure 3 shows the strength development of specimens with varying lime dosages at different ages. Under curing conditions of 30 °C and 30% relative humidity, strength increased with age, with rapid growth in the early stages followed by slower growth later on. For specimens cured for less than 28 days, higher lime dosages generally resulted in higher strength. Notably, the specimen with 5% lime showed a significant increase in late strength, reaching 10.54 MPa at 180 days, which is much higher than the other dosages. This suggests that lime dosage does not necessarily correlate with late strength, as excessive lime may limit further strength development. The reasons for this will be explored in the following micro-analysis.

#### 2.2.2. XRD Analysis

**Figure 4:**
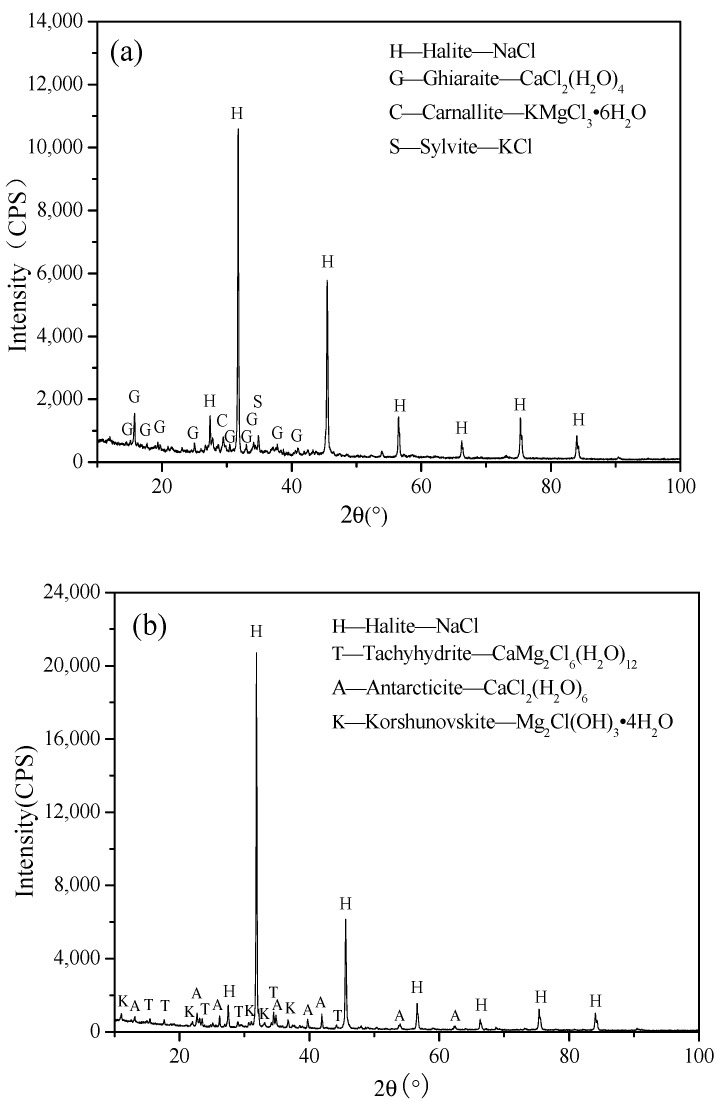

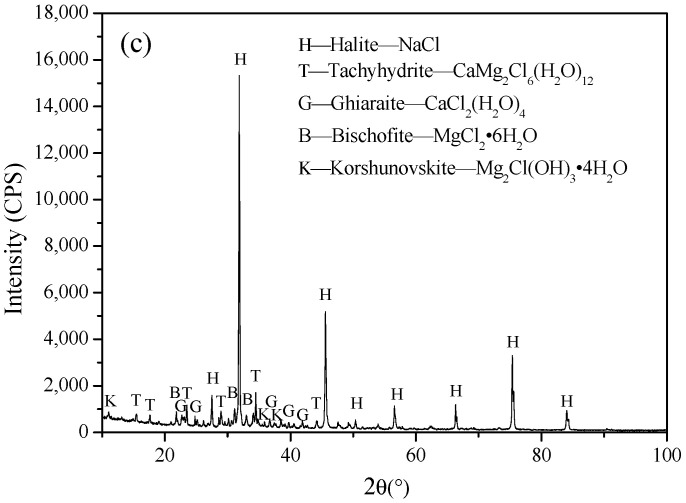
XRD patterns of hydration products of filling materials with different ratios after 180 days of maintenance. (**a**) 9% lime; (**b**) 5%; (**c**) 2.6%.

From Figure 4, it is obvious that sodium chloride crystals are the main chemical component in the XRD patterns of the three test blocks, due to the fact that the potash mine tailing salt acts as an aggregate in the system. From the compositions other than NaCl, it can be seen that the hydration products corresponding to different lime dosages are not the same. The hydration products in the specimen with 9% lime are mainly ghiaraite (CaCl_2_(H_2_O)_4_) and carnallite (KMgCl_3_·6H_2_O). However, the hydration products in the specimen with 5% lime include antarcticite (CaCl_2_(H_2_O)_6_), tachyhydrite (CaMg_2_Cl_6_(H_2_O)_12_), and korshunovskite (Mg_2_Cl(OH)_3_·4H_2_O). For 2.6% lime dosage, the products are mainly tachyhydrite (CaMg_2_Cl_6_(H_2_O)_12_), ghiaraite (CaCl_2_(H_2_O)_4_), bischofite (MgCl_2_·6H_2_O), and korshunovskite (Mg_2_Cl(OH)_3_·4H_2_O), etc.

Figure 4 clearly shows that sodium chloride crystals dominate the XRD patterns of all three test blocks, as the potash mine tailing salt acts as an aggregate in the system. The hydration products vary with different lime dosages. In the 9% lime specimen, the main hydration products are ghiaraite (CaCl_2_·4H_2_O) and carnallite (KMgCl_3_·6H_2_O). The 5% lime specimen primarily contains antarcticite (CaCl_2_·6H_2_O), tachyhydrite (CaMg_2_Cl_6_·12H_2_O), and korshunovskite (Mg_2_Cl(OH)_3_·4H_2_O). For the 2.6% lime dosage, the products are mainly tachyhydrite, ghiaraite, bischofite (MgCl_2_·6H_2_O), and korshunovskite.

When lime is added to the brine, it initially reacts with water to form Ca(OH)_2_, which consumes the solution’s water. This then reacts with MgCl_2_ to produce Mg(OH)_2_ and CaCl_2_. The CaCl_2_ readily forms hydrated calcium chloride compounds like ghiaraite and antarcticite. In the 9% lime specimen, more water is consumed, resulting in greater calcium chloride hydrate formation. As a result, the MgCl_2_ in the system reaches saturation faster, leading to the formation of magnesium chloride hydrates such as bischofite or the precipitation of carnallite with KCl. With lower lime dosages (5% and 2.6%), less hydrated calcium chloride is formed, but tachyhydrite, bischofite, and korshunovskite appear in greater amounts.

CaO (s) + H_2_O (l) + MgCl_2_ (aq) = Mg(OH)_2_ (s) + CaCl_2_ (aq) (1)

CaCl_2_ (aq) + 2MgCl_2_ (aq) + 12H_2_O (l) = CaMg_2_Cl_6_ (H_2_O)_12_ (s) (2)

MgCl_2_ (aq) + 6H_2_O (l) = MgCl_2_·6H_2_O (s) (3)

3Mg(OH)_2_ (s) + MgCl_2_ (aq) + 8H_2_O (l) = 3Mg(OH)_2_·MgCl_2_·8H_2_O (s) (4)

After 180 days of curing, the cementation in the test block with 9% lime doping was weaker than in the block with 5% lime doping. The crushed surface of the 9% lime block resembled a powder, likely due to the poor water retention of hydrated calcium chloride. In contrast, the 5% lime block showed a stronger bond on the crushed surface. Further microscopic analysis will be conducted using SEM.

#### 2.2.3. Microscopic Morphology

**Figure 5:**
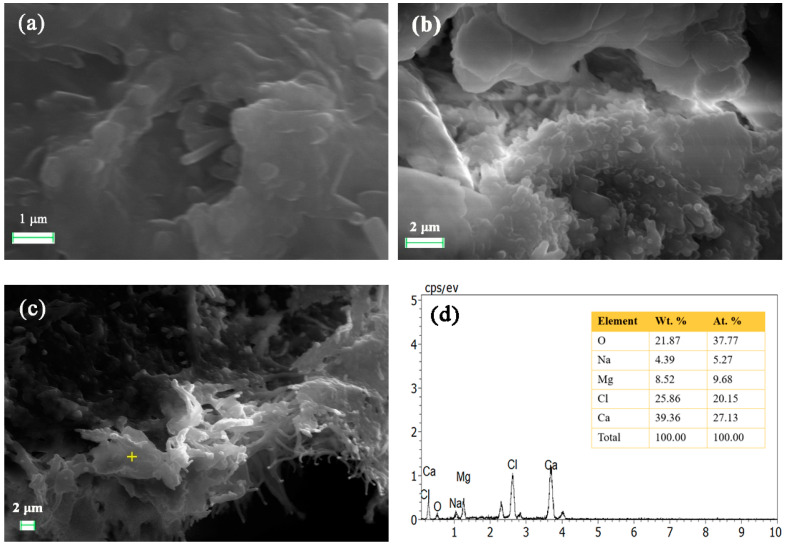
(**a**–**c**) SEM images of 180-day test blocks with 9% lime doping and (**d**) the EDS result of the crisscross point in (**c**).

Figure 5 shows the SEM images and electron spectra of 180-day test blocks with 9% lime doping. In Figure 5a, a relatively complete columnar crystalline structure is observed in the 9% lime system. However, the crystals are weakly bonded to other hydration products and aggregates, preventing the formation of a compact structure. In Figure 5b,c, the columnar hydrate crystals and gelatinous matrix exhibit a tendency to interpenetrate and wrap, which positively affects the strength development of the filling material. However, lime’s significant volume shrinkage creates large gaps between the crystals, hindering further strength growth. As a result, the strength development of the 9% lime system is slower than that of the 5% lime system, and its 180-day strength is notably lower. Figure 5d shows that the crystals primarily consist of hydrated calcium chloride.

**Figure 6:**
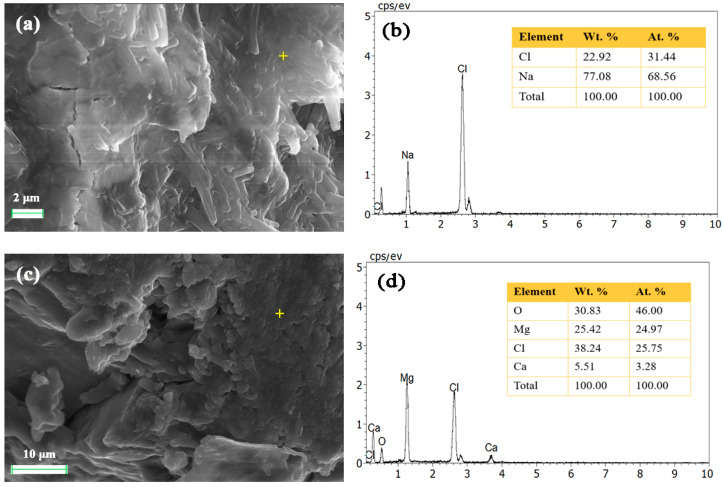
SEM images of 180-day test blocks with 5% lime doping ((**a**) and (**c**)) and the corresponding EDS results of the crisscross points ((**b**) and (**d**), respectively).

Figure 6 shows the SEM images of 180-day test blocks with 5% lime doping and the electron spectra. From Figure 6a,c, it can be seen that the overall structure is relatively dense, the porosity is very low, and the hydrate is tightly bonded to the matrix as a whole, which is the reason why this test block can reach 10 MPa. In this test block, the lime doping is 5%; it will not cause large porosity due to the volume contraction caused by too much lime doping, nor will it be difficult to harden due to too little lime doping to play the role of cementation, so in the three different ratios, this lime doping should be corresponded to the highest long-term strength of the test block.

As shown in Table 1, the −100 mesh fraction of the tailing salt powder is 24.23%, suggesting that the gelatinous matrix responsible for wrapping the hydrate may result from the recrystallization of fine tailing salt powder. The dense microstructure in Figure 6a is from the recrystallization of NaCl, from the EDS result in Figure 6b. This indicates that sodium chloride plays a role in promoting strength growth. One fact to support this theory in our study is that test blocks made with only lime and brine fail to achieve high strength and are difficult to demold, whereas mixing brine with tailing salt leads to recrystallization and increased strength. These results confirm that tailing salt plays a crucial role in enhancing the performance of lime-based gels in this system. Other researchers [19,20,21] also found that caking of sodium chloride can conduct at ambient relative humidity because of dissolution and recrystallization process. Similarly, Fliß et al. [22] indicated that the strong backfilling structure forms from the three-axial stress caused by the convergence of backfilled areas, leading to recrystallization and compaction of rock salt grains with new binding cement.

**Figure 7:**
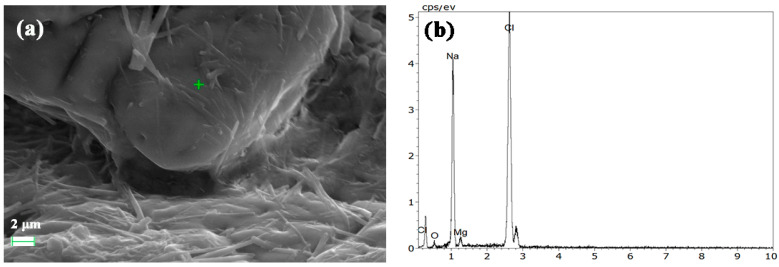

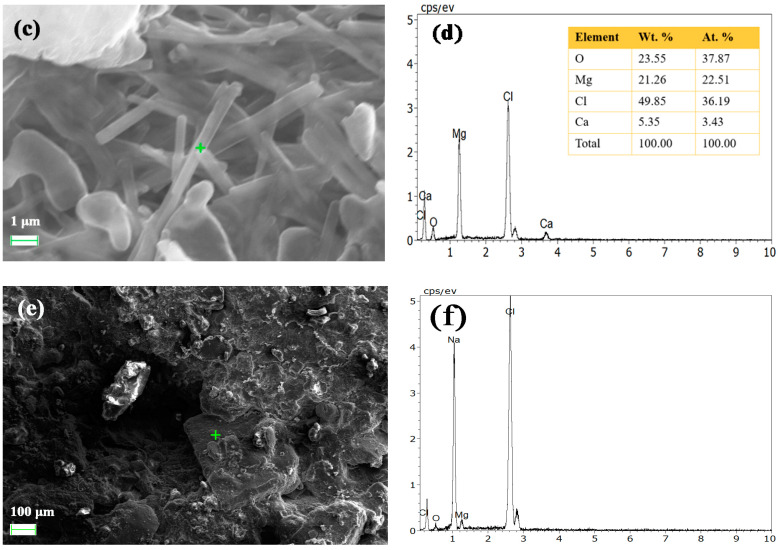
SEM images of 180-day test blocks with 2.6% lime doping ((**a**), (**c**) and (**e**)) and the corresponding EDS results of the crisscross points ((**b**), (**d**), and (**f**), respectively).

Figure 7 shows SEM images and electron spectra of 180-day test blocks with 2.6% lime doping. From Figure 7a,c,e, the overall structure appears loose, with columnar hydrates not fully integrating with the matrix and increased porosity between particles. These factors hinder strength development. This behavior may be attributed to the reduced water retention caused by the low lime content, which limits recrystallization of the tailing salt and results in insufficient hydrate formation. The electron spectra reveal a clear dependence of the hydration product composition on lime dosage: higher lime content increases Ca levels, while lower lime content leads to higher Mg content. This aligns with XRD results showing different hydrate types for varying lime dosages.

By comparing Figure 5b, Figure 6a, and Figure 7a, which are nearly uniformly magnified, it can be indicated that, among the three samples, L5 exhibits the most compact microstructure, with aggregates and hydration products tightly bonded together. In contrast, L2.6 and L9 have relatively loose structures with weaker connections between materials.

#### 2.2.4. Thermogravimetric Analysis

**Figure 8:**
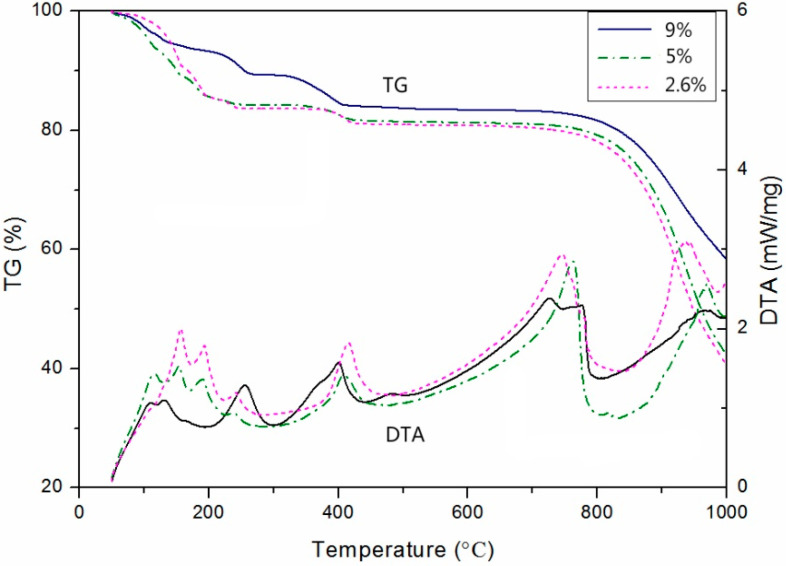
Thermogravimetric analysis of 180-day hydration products of specimen blocks with different lime dosages.

Figure 8 shows the TG/DTA analysis of hydration products over 180 days for different lime dosages. The thermogravimetric curves for the three specimens vary, reflecting differences in the substances formed, which aligns with the XRD results.

Between 50 and 300 °C, dehydration and dehydrochlorination reactions occur. For instance, bischofite (MgCl_2_·6H_2_O) loses two water molecules to form magnesium chloride tetrahydrate (MgCl_2_·4H_2_O) (96–117 °C), then loses two more to form calcium chloride dihydrate (135–180 °C). Between 185 and 230 °C, one water molecule is lost to form magnesium chloride monohydrate, or one molecule of HCl is released to produce Mg(OH)Cl [23]. The endothermic peak around 400 °C corresponds to the removal of remaining water and the volatilization of HCl, while hydromagnesite transforms into magnesite. The DTA peaks between 600 and 800 °C represent the melting of chlorides. The melting points of NaCl, KCl, and CaCl_2_ are 801 °C, 770 °C, and 782 °C, respectively, which explains the volatilization of chlorides above 800 °C and the corresponding decrease in the TG curves.

#### 2.2.5. Infrared Analysis

**Figure 9:**
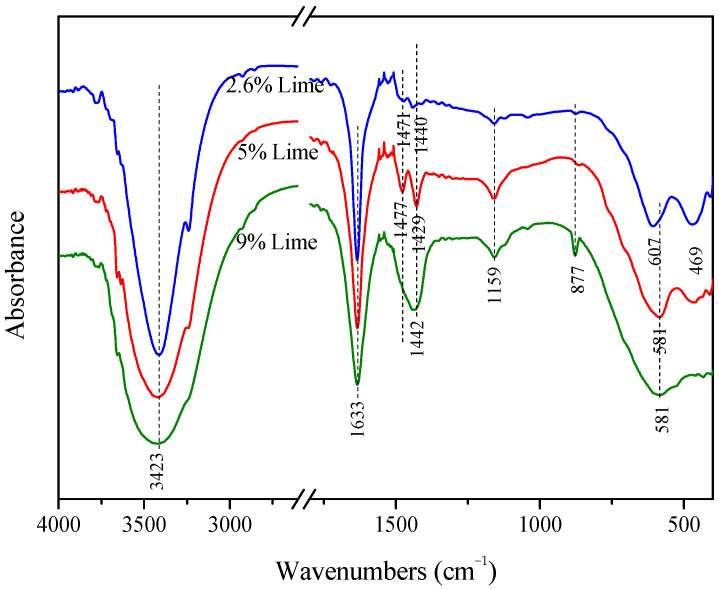
Infrared analysis of 180-d hydration products of specimens with different lime dosage.

Figure 9 shows the FTIR analysis of 180-day test blocks with different lime dosages, and it can be seen that the curve trends are basically the same, which is due to the fact that there is no absorption band for Cl^-^ at 400~4000 cm^−1^, and the difference between the three different lime dosages lies in the difference in the metal elements that form ionic bonds with chlorine. The absorption bands at 3423 cm^−1^ and 1633 cm^−1^ are the O-H stretching and bending vibration bands in the water molecules of the hydration products, respectively, and the appearance of the extended bands is attributed to the formation of hydrogen bonding with a wider range of strengths. The absorption bands at 1477 cm^−1^, 1471 cm^−1^, 1442 cm^−1^, 1429 cm^−1^, 1440 cm^−1^, and 877 cm ^−1^ indicate asymmetric stretching and bending vibration bands in the CO_3_^2−^, respectively. The vibration bands of CO_3_^2−^ appeared in all the samples, which indicates that the lime carbonation reaction occurred. It can be seen that, with the increase in lime doping, the width and depth of the CO_3_^2−^ vibration bands of the generated specimens increased, which indicates that more calcium carbonate was generated.

## 3. Conclusions

Lime is mainly used as a gel for potash mine filler in this study, with fluidity and strength properties being investigated at varying lime dosages and solid concentration. Besides, with the solid concentration set at 55% and the lime dosage at 9%, 5%, and 2.6%, respectively, the generated hydration products and the corresponding microstructures are studied, and the effects of lime dosage on the mechanical properties of the filler are explored. The main conclusions are as follows:

(1) When lime is used as a gel for underground filling material in potash mines, to satisfy both the flow and strength requirements of the filling material, for lime content of 0.5–1%, the solid concentration should be 70–75%; for 1–2%, it should be 60–65%; and for 2–3%, it can be 55% or below. Using lime as a gel enhances resource availability, and lowers environmental carbon emissions, making it a cost-effective and eco-friendly choice.
(2) The hydration products in the 180-day test block with 9% lime dosing are mainly ghiaraite (CaCl_2_·4H_2_O) and carnallite (KMgCl_3_·6H_2_O). With 5% lime (L5), tachyhydrite (CaMg_2_Cl_6_·12H_2_O) predominates, along with minor amounts of antarcticite (CaCl_2_·6H_2_O) and korshunovskite (Mg_2_Cl(OH)_3_·4H_2_O), and with 2.6% lime (L2.6), the products include tachyhydrite, ghiaraite, bischofite (MgCl_2_·6H_2_O), and korshunovskite.
(3) The long-term strength of lime is not proportional to the lime dosage, and the 180-day compressive strength of L9 is much lower than L5, which is due to the large volume contraction in the hardening process of lime and the formation of voids in the specimen with excessive lime dosage, which hinders the further improvement of strength.
(4) When the lime dosing is 5% and the solid concentration is 55%, it will neither cause large porosity due to volume contraction caused by too much lime dosing, nor will hardening be difficult due to too little lime dosing to play the role of cementation, so this ratio has the highest long-term strength of the corresponding test block in the three tests.
(5) The −100 mesh of the tail salt powder after grinding is 24.23%, so it is possible that the gelatinous matrix that plays a role in wrapping the hydrate is the product of the recrystallization of the fine powder of the tail salt. It can be seen that, in this system, sodium chloride plays a certain role in promoting the growth of strength.

## 4. Materials and Methods

### 4.1. Materials

#### 4.1.1. Salt Tailings

**Figure 10:**
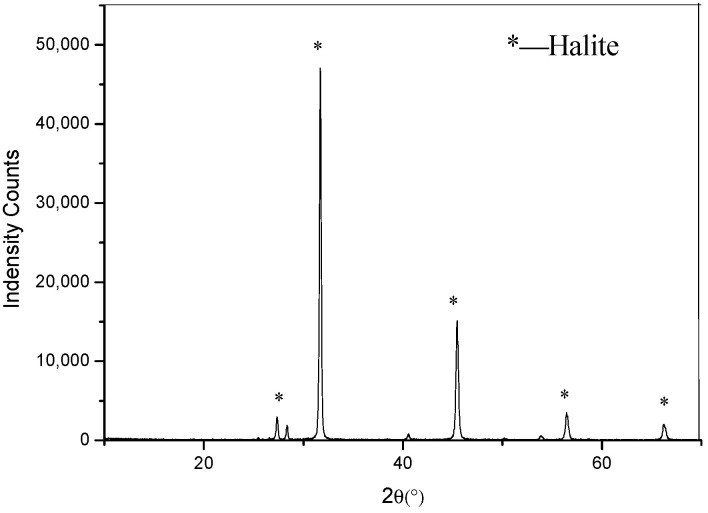
XRD analysis of tailing salt.

The potash tailing salt used in the ring tube test is a granular, colorless to milky white (off-white when impure) material, with a NaCl content over 95% (Figure 10) and a maximum particle size of 9 mm. It serves as the aggregate in the filling material, with its original particle size distribution listed in Table 2.

The uneven particle size distribution of the tailing salt causes significant segregation when used directly as aggregate. To address this, the coarse-grained tailing salt (+2.5 mm) is ground for 10 min using an SM φ500 × 500 mini-mill and then blended with the other raw materials. This process improves particle packing and enhances the workability of the filling material. The final particle size distribution of the tailing salt is shown in Table 1.

#### 4.1.2. Brine Water

The potash mine brine was colorless when pure but turned dark yellow with certain impurities. It had a density of 1.25 g/cm^3^ and a total solute concentration of 36.93%. Elemental analysis of the latest shipment’s tailings was conducted using an inductively coupled plasma emission spectrometer (PerkinElmer Optima 8300 Series), with solution compositions provided in Table 3.

#### 4.1.3. Lime

**Figure 11:**
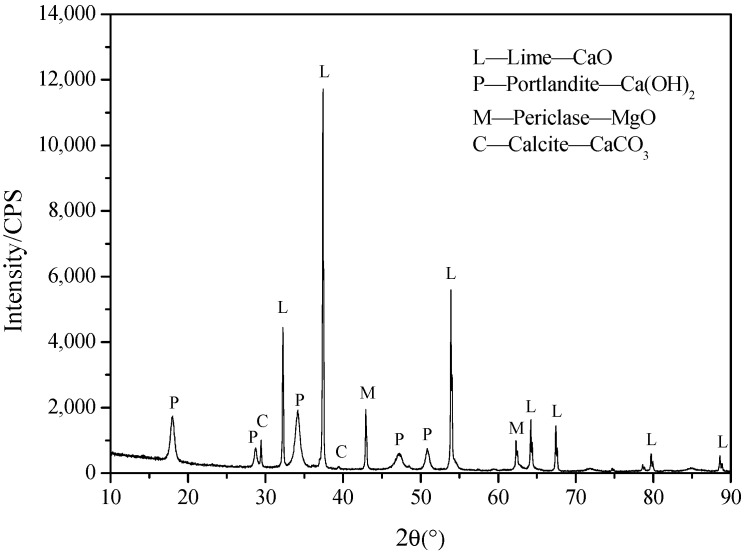
XRD pattern of calcined and ground lime.

The lime raw material used in the test was locally quarried limestone from Laos, with its chemical composition shown in Table 4. The limestone was crushed to −20 mm, then calcined at 1100 °C for 12 h. After calcination, the lime was ground in an SM φ500 × 500 test mill for 5 min to produce a gel for potash mine CPBs. The XRD pattern of the calcined and ground lime (Figure 11) revealed the main minerals as lime (CaO), portlandite (Ca(OH)_2_), periclase (MgO), and calcite (CaCO_3_). The particle size distribution is shown in Table 5.

### 4.2. Methods

#### 4.2.1. Preparation of CPBs with Varying Lime Dosages and Solid Concentrations

When lime is used as a gel in filling materials, the amount of lime significantly influences both the slurry’s flow properties and the strength of the hardened material. Tests have shown that even small adjustments (0.5% or less) in lime dosage can cause substantial changes in performance. Therefore, it is crucial to study the effect of lime dosage on these properties. This test was designed to ensure the prepared filling slurry maintains a flowability above 200 mm within 1 h, and that the compressive strength of the hardened material exceeds 1 MPa after 28 days of curing.

The lime dosage test ratio is shown in Table 6. The selected lime gel has a fineness of −100 mesh, comprising 55%. The test ratio was designed based on previous experiments and practical experience, which indicate that the key factor influencing lime dosage is the solid concentration of the filling material. At low solid concentrations, more lime is required to prevent issues such as slurry segregation and water secretion, while ensuring the hardened material achieves the desired strength. Conversely, at high solid concentrations, the lime dosage should be reduced to avoid rapid setting, which could impede pipeline transport.

When the lime dosage is less than 0.5%, even with a solid concentration of 70%, the compressive strength of the 28-day test block will not reach 1 MPa. If the solid concentration drops to 55%, the lime dosage must be increased above 2.5% to prevent segregation. However, when the dosage exceeds 2.5%, the slurry will set too quickly, losing fluidity within an hour. Therefore, lime dosages should be kept between 1 and 2.5% for low solid concentrations (below 60%), between 0.5 and 2% for medium concentrations (60–70%), and not exceed 2.5% for high concentrations (above 70%). Under high solid concentration conditions (≥70%), the lime dosage should range from 0.5 to 1.5%.

Lime, tailing salt, and brine water are mixed according to Table 6 to prepare the CPB slurry, with fluidity measured every 0.5 h for the first 2 h. The slurry is then poured into 40 mm × 40 mm × 160 mm molds and placed in a curing chamber set to 30% relative humidity and 40 °C, simulating conditions in a potash mine goaf. After removing the molds, the CPBs undergo strength testing at different curing ages.

#### 4.2.2. Preparation of Pastes

To investigate the hydration mechanism of lime as a gel for potash mine filling material, test blocks were prepared using lime, potash mine tailing salt, and potash mine tailing liquid according to the ratios in Table 7. The solid concentration was set at 45%, with lime content at 9%, 5%, and 2.6%, respectively, to examine the impact of lime proportions on the system’s strength and product composition. Lime and potash mine tailing salt were thoroughly mixed and combined with potash mine tailing liquid in an NJ-160A cement mortar mixer. The mixture was then poured into 3 cm × 3 cm × 5 cm cement mortar molds, vibrated on a cement sand adhesive vibration table, and cured at 40 °C and 30% humidity. After curing for the specified period, the molds were removed, and the compressive strength was measured.

#### 4.2.3. Strength Testing and Microanalysis

The compressive strength was measured using a YES-300 digital hydraulic pressure tester from Changchun First Material Testing Machine Factory. Flowability of the filling material was determined following GB/T 2419-2016 [24]. Microanalyses of the net paste samples included X-ray diffraction (XRD), scanning electron microscopy with energy-dispersive spectroscopy (SEM-EDS), simultaneous thermal analysis (TG-DTA), and Fourier-transform infrared (FTIR) spectroscopy. XRD was used to assess the mineral composition and crystallinity of the hydration-hardened slurry, with spectra obtained on a Rigaku Ultima IV diffractometer at the School of Materials, University of Science and Technology Beijing. SEM was employed to observe the microstructure, while EDS helped identify the physical phases, with data collected at the State Key Laboratory of New Metallic Materials, University of Science and Technology Beijing, using a Cambridge S250 SEM and LinkNA1000 energy spectrometer. Thermal stability and composition of the hydration products were analyzed using a NETZSCH STA 449F3 thermal analyzer at Tsinghua University, with a temperature range of 50–1000 °C. FTIR analysis of the hydrated products was performed with a NEXUS-670 spectrometer, operating at a resolution of 3 cm^−1^ and a wavenumber range of 400–4000 cm^−1^.

## Figures and Tables

**Table 1:** Final tailings salt aggregate size distributions.

Size Range/mm	−4 + 2.5	−2.5 + 0.63	−0.63 + 0.3	−0.3 + 0.25	−0.25 + 0.15	−0.15
Percent/%	1.6	37.44	22.65	5.36	8.72	24.23

**Table 2:** Particle size analysis of raw tailing salt.

Size Range/mm	+5	−5 + 4	−4 + 2.5	−2.5 + 0.63	−0.63 + 0.3	−0.3 + 0.25	−0.25 + 0.15	−0.15
Percent/%	15.5	8.5	24.3	30.3	17.0	1.3	2.6	0.8

**Table 3:** Solute composition content of the brine water.

Solute	MgCl_2_	KCl	NaCl	CaCl_2_	Total
Concentration/%	34.78	0.85	1.12	0.18	36.93

**Table 4:** Lime chemical compositions.

Chemical Compositions	SiO_2_	CaO	K_2_O	Fe_2_O_3_	Al_2_O_3_	Loss
Content/%	0.10	41.98	0.02	0.05	0.02	57.83

**Table 5:** Particle size distribution of calcined lime after grinding.

Particle Size/μm	+150	−150 + 74	−74
Content/%	51.56	25.21	23.23

**Table 6:** Mix proportion of CPBs with different lime dosages and solid concentrations.

No.	Lime/%	Solid Concentration/%	Tailing Salt/%	Brine Water/%
1	1	55	54	45
2	1.5		53.5	
3	2		53	
4	2.5		52.5	
5	3		52	
6	0.5	60	59.5	40
7	1		59	
8	1.5		58.5	
9	2		58	
10	0.5	65	64.5	35
11	1		64	
12	1.5		63.5	
13	2		63	
14	0.5	70	69.5	30
15	1		69	
16	1.5		68.5	
17	0.5	75	74.5	25
18	1		74	
19	1.5		73.5	

**Table 7:** Test block raw material ratio.

No.	Lime/%	Tail Salt/%		Brine Water/%
		+2.5 mm (Milled for 5 min)	−2.5 mm	
L9	9	18	18	55
L5	5	20	20	55
L2.6	2.6	21.2	21.2	55

## Data Availability

The original contributions presented in this study are included in the article. Further inquiries can be directed to the corresponding authors.
